# New approach in evaluation of ceramic-polymer composite bioactivity and biocompatibility

**DOI:** 10.1007/s00216-017-0518-0

**Published:** 2017-07-26

**Authors:** Leszek Borkowski, Anna Sroka-Bartnicka, Izabela Polkowska, Marta Pawlowska, Krzysztof Palka, Emil Zieba, Anna Slosarczyk, Krzysztof Jozwiak, Grazyna Ginalska

**Affiliations:** 1 Chair and Department of Biochemistry and Biotechnology, Medical University of Lublin, Chodzki 1, 20-093 Lublin, Poland; 2 Department of Biopharmacy, Medical University of Lublin, Chodzki 4a, 20-093 Lublin, Poland; 3 Department and Clinic of Animal Surgery, University of Life Sciences in Lublin, Gleboka 30, 20-612 Lublin, Poland; 4 Department of Animal Physiology, University of Life Sciences in Lublin, Akademicka 12, 20-033 Lublin, Poland; 5 Department of Materials Engineering, Lublin University of Technology, Nadbystrzycka 36, 20-618 Lublin, Poland; 6 SEM Laboratory, Department of Zoology and Ecology, John Paul II Catholic University of Lublin, Al. Krasnicka 102, 20-718 Lublin, Poland; 7 Faculty of Materials Science and Ceramics, AGH-University of Science and Technology, Mickiewicza 30, 30-059 Krakow, Poland

**Keywords:** Bioactivity, Biomaterials, Bone substitutes, Carbonate hydroxyapatite, Mineralization, Raman spectroscopy, SEM

## Abstract

Regeneration of bone defects was promoted by a novel β-glucan/carbonate hydroxyapatite composite and characterized by Raman spectroscopy, microCT and electron microscopy. The elastic biomaterial with an apatite-forming ability was developed for bone tissue engineering and implanted into the critical-size defects of rabbits’ tibiae. The bone repair process was analyzed on non-decalcified bone/implant sections during a 6-month regeneration period. Using spectroscopic methods, we were able to determine the presence of amides, lipids and assign the areas of newly formed bone tissue. Raman spectroscopy was also used to assess the chemical changes in the composite before and after the implantation process. SEM analyses showed the mineralization degree in the defect area and that the gap size decreased significantly. Microscopic images revealed that the implant debris were interconnected to the poorly mineralized inner side of a new bone tissue. Our study demonstrated that the composite may serve as a biocompatible background for collagen ingrowth and exhibits the advantages of applying Raman spectroscopy, SEM and microCT in studying these samples.

**Electronic supplementary material:**

The online version of this article (doi:10.1007/s00216-017-0518-0) contains supplementary material, which is available to authorized users.

## Introduction

The increasing number of accidents, injuries and bone tumours along with developments in medical sciences result in growing demand for bone substitute materials. The global bone grafts and substitutes (BGS) market was valued at US$2358.3 million in 2014 and is expected to reach US$3482.0 million by 2023 according to a new report published by Transparency Market Research [1]. Plenty of scientific reports concerning novel materials appear every year and regard many different aspects such as material properties, scaffold design, host response, implant personalization, use of cells and signalling molecules [2, 3]. Ceramic-polymer composites containing synthetic hydroxyapatite have received much attention because of their advantageous properties such as biocompatibility, adaptation to the shape/size of bone defects, sufficient mechanical strength, non-toxicity and possibility of delivering drugs and macromolecules [4–10].

In vivo evaluation of a new bone substitute material in an animal model usually include radiographic examination [11], densitometry (BMC and BMD parameters) [12, 13], histological analysis [14], histomorphometry [15, 16], biochemistry [17], microhardness [18], computed tomography [19, 20], compressive test [21] and electron micrography [22]. Recently, Raman spectroscopy (widely used in chemistry) has also found application in the assessment of bone quality. This technique has become a valuable tool in bone implant testing [23] and bone tissue characterization [24, 25]. It allows understanding how changes in bone composition and structure influence tissue-level mechanical properties. The information about the mineral and matrix collagen components, bone crystallinity, bone hardness, orientation of mineral crystallites, age of the tissue but also indirect information on collagen cross-links [26–28]. The technique can be used with fresh, as well as fixed and imbedded, specimens and in some limitation for non-invasive measurements on live animals [29]. The Raman spectroscopy has been used in various studies for the analysis of soft tissues without sample preparation and to obtain new and complementary information about biominerals [30].

In this study, we propose a new approach in evaluation of bone implants bioactivity and biocompatibility by the use of a combination of different techniques—Raman spectroscopy, high-resolution X-ray microtomography (microCT) and scanning electron microscopy (SEM). The combination of these non-invasive techniques was applied to study chemical changes in the material before and after implantation, bone implant integration, mineralization degree in different points within defect site (“bone maturation”), collagen ingrowth and samples surface in micro- and macro-scale. The subject of research was novel ceramic-polymer composite dedicated for bone tissue engineering consisting of carbonate-substituted hydroxyapatite (CHAP) and polysaccharide (β-1,3-glucan). Regenerative capability, bioactivity and osteoconductivity of the composite were tested in an animal model and examined qualitatively.

## Experimental

### Sample preparation

CHAP granules were synthesized at the AGH-University of Science and Technology, according to patented procedures [31, 32]. β-1,3-Glucan (curdlan) from *Alcaligenes faecalis* (DP 450) was supplied by Wako Chemicals, Japan.

CHAP-glucan composite scaffolds were prepared according to the procedure described in European patent [33]. Briefly, samples were fabricated by mixing 3 g of CHAP granules with β-1,3-glucan aqueous suspension (0.625 g of glucan + 5 ml of distilled water), thus obtaining wt% proportion granules to β-1,3-glucan 83:17. The mixture of ceramic/glucan solution was put into a special glass mould to obtain cylindrical implants, 4 mm in diameter and 6 mm in length and heat treated at 90 °C for 15 min. Finally, the fabricated material was dried for 4 days at 37 °C, subjected to exsiccation for the next 3 days and sterilized. Table 1 summarizes properties of the CHAP-glucan composite.

**Table 1:** Chemical compositions and physical parameters of the CHAP-glucan composite used in the study

Characteristics	Composites CHAP/glucan
CHAP/glucan (wt% ratio)	83:17
Carbonate content (wt%)	4.3
Sorption index (%)	119
Compressive strength (MPa)^a^	6.1
Young’s modulus (GPa)^a^	0.64

^a^Measured for dry composite samples

### Implantation procedure

**Fig. 1:**
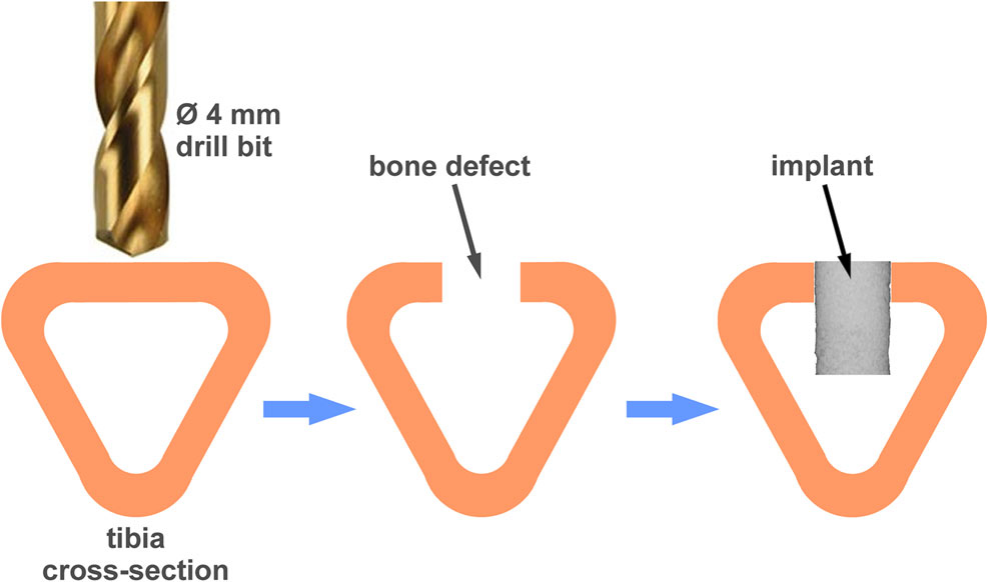
Implantation procedure illustrated on a rabbit tibia cross-section. A defect 4 mm in diameter was drilled 2.5–3 cm below the epiphyseal cartilage. The cavities were filled with the CHAP-glucan composite material in the form of cylindrical implants, 4 mm in diameter and 6 mm in length

With approval of the II Local Ethics Committee on Animal Research University of Life Sciences in Lublin, Poland (agreement no 16/2010), 24 New Zealand male white rabbits (6 months old, c.a. 3.5 kg body mass) were used in the experiment. Animals were kept separately in cages under standard conditions of housing, feeding and handling. The adaptation at the animal house of the Medical University of Lublin lasted 1 week. Prior to surgery, animals were premedicated with medetomidine (Domitor®, Orion Corp., Finland; dose 0.5 mg/kg) and anesthetized with ketamine (Bioketan®, Biowet, Poland; 0.5 mg/kg). Surgical procedure was described previously by Borkowski et al. [34, 35]. Briefly, a defect, 4 mm in diameter and 6 mm in depth, was drilled in the proximal tibial metaphysis and filled with CHAP-glucan composite material. The skin was sutured with Dexon 3–0 thread (Tyco Healthcare, UK). The implantation procedure has been shown in Fig. 1. After the operation, animals were allowed to move freely in cages.

Rabbits were euthanized 1, 3 and 6 months after composite implantation with an overdose of sodium pentobarbital (Morbital®, Biowet, Poland; 1 ml/kg). The isolated tibiae (8n each group) from all three time points were frozen in liquid nitrogen and stored at −20 °C until all samples were collected. For Raman and SEM analysis, 2-mm sections were collected from undecalcified bones using a diamond-edged stainless steel rotary dental saw for the 6-month group.

### Raman spectroscopy

Raman spectra were collected using a DXR Raman Microscope (Thermo Scientific, Waltham, MA, USA), with a 780-nm laser with a maximum output power of 20 mW. The spectra were recorded over the spectral range of 2500–200 cm^−1^ using an operating spectral resolution of 4 cm^−1^ of Raman shift. The 25 square aperture was used with the following settings: exposure time 6 s, number of exposures 8. The spectra were collected using both a ×10 and a ×100 objective. All data processing and image assembly were performed using OMNIC 8.2.0.387 software. The five spectra from the each area in the sample were collected then averaged. The spectra were baseline corrected in the range 2000–200 cm^−1^, not normalized and present relative intensity of the bands. The autofocus at each measured point of sample was used in case of non-flat samples. The distance between five marked points in the Fig. 5d was 300 μm, and the total distance was about 1500 μm.

### MicroCT and SEM analysis

Bones with implanted composite were scanned in wet state using Skyscan 1172 X-ray computed tomography (Bruker microCT, Belgium). The sample, placed in tube made of polyethylene (PE) filled with distilled water, was rotated during scanning within the angular range of 0°÷180° with a step of 0.2°. An averaging mode was used to obtain balanced exposure levels. In total, 900 photographs were made. An aluminium 0.5-mm-thick filter was used to reduce beam hardening effects. To eliminate possible artefacts, the sample was randomly moved for each projection. The set of images were then reconstructed into cross-sections using the NRecon software (Bruker microCT, Belgium). After the reconstruction, the isotropic voxel size was 3.94 μm in each axis. Imaging of results was made in DataViewer software (Bruker microCT, Belgium) using orthogonal sections and transforming grey scale into colours to easy recognize objects.

The bone implant sections were analysed using scanning electron microscopy (FE-SEM; Zeiss ULTRA plus).

## Results and discussion

**Fig. 2:**
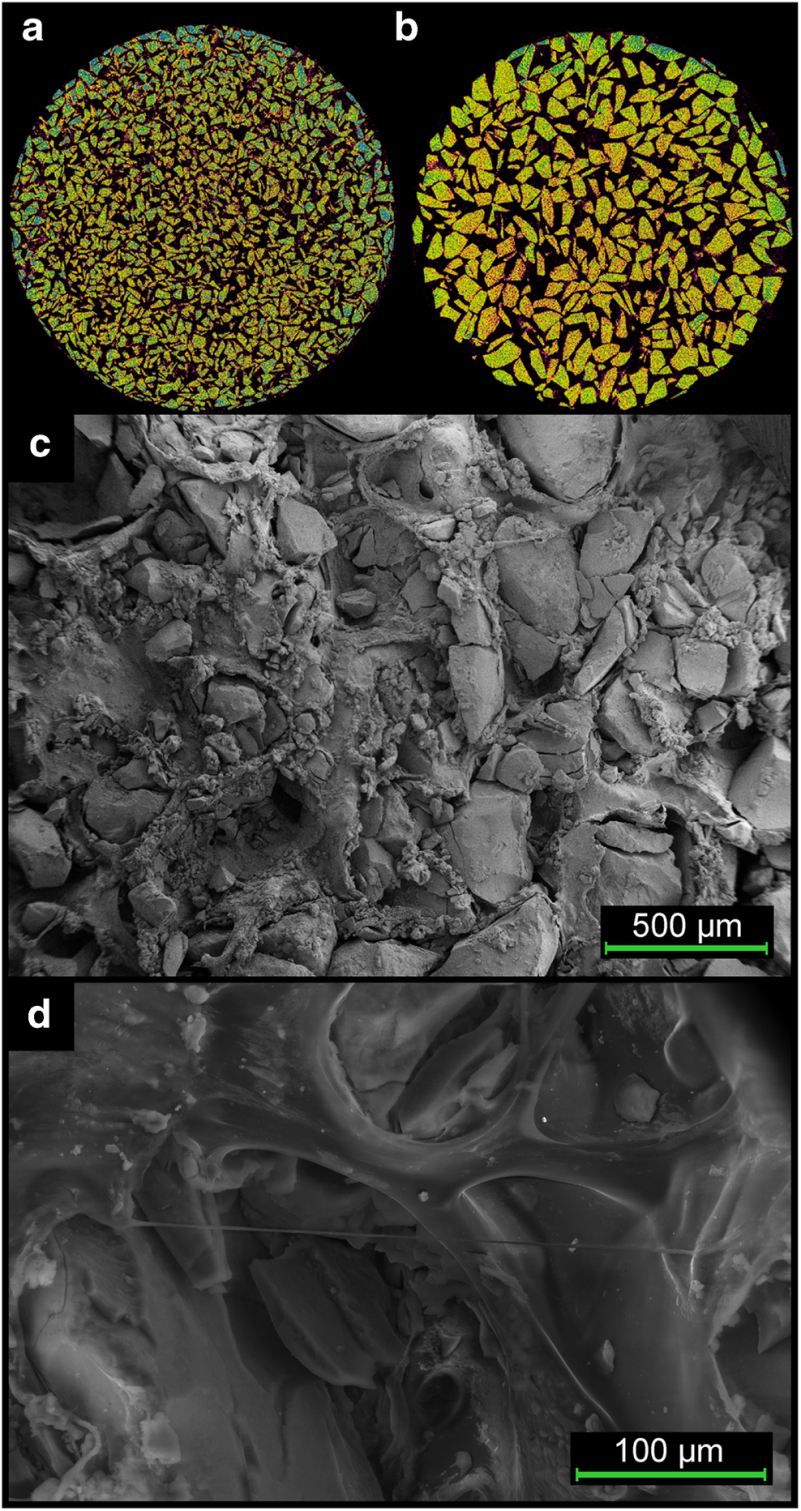
MicroCT images of CHAP granules and SEM images presenting structure of CHAP/glucan biocomposite. (**a**) 0.2–0.3 mm fraction of CHAP granules, (**b**) 0.4–0.6 mm fraction of CHAP granules, (**c**) CHAP/glucan composite surface magnified 120× and (**d**) scaffolds microarchitecture magnified ×500

Figure 2a, b demonstrated irregular shapes of the synthesized CHAP granules and uniform density of the particles among fractions. Composite scaffolds were obtained after addition of β-1,3-glucan, which formed a compact mesh widely adhered to CHAP surface (Fig. 2c). Scaffold microarchitecture shown in Fig. 2d exhibited a high porosity and opened pore structure. SEM images also confirmed a homogenous distribution of granules in the scaffolds. Composite samples exhibited high flexibility, could be compressed or bent and adapted easily to appropriate shapes. The obtained material could be formed into different shapes at the preparation step (using different moulds) and after fabrication using scissors or lancet.

### Raman spectroscopy

To investigate the chemical changes in the bone tissue regeneration process, we focused on sample that were implanted for 6 months in the area between the bone tissue and implanted composite. Raman spectroscopy was chosen as a non-destructive technique that requires minimal sample preparation [36] and that can be used to measure the chemical properties of the mineral and collagen parts. The Raman spectrometer was coupled with microscope optics for sample illumination and spectral acquisition, allowing investigation at the micron level [37].

**Fig. 3:**
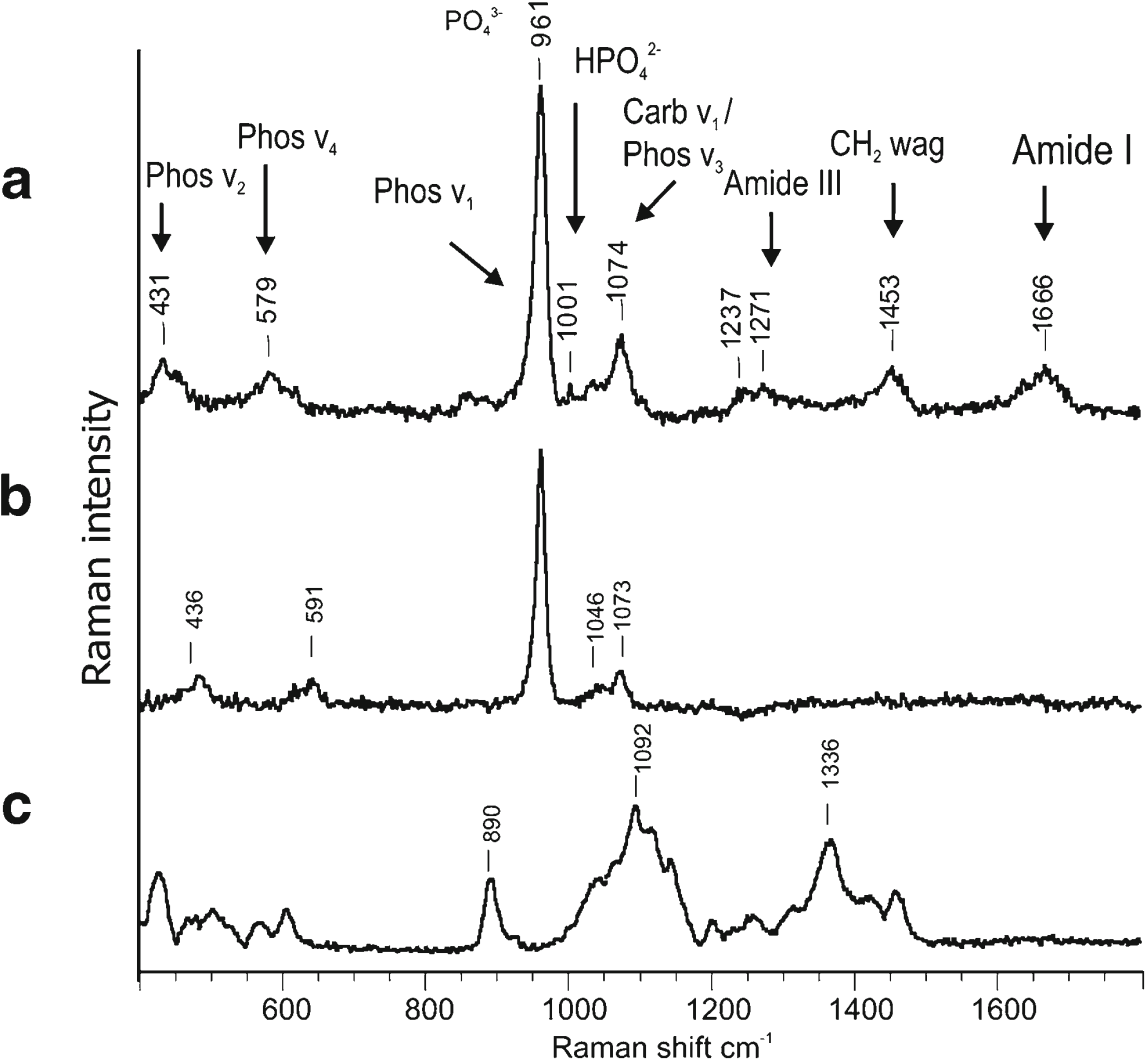
The Raman spectra of representative bone tissue sample (**a**) and composite material components before implantation: CHAP granule (**b**) and β-1,3-glucan (**c**)

A representative Raman spectrum of bone is shown in Fig. 3a. The band at 960 cm^−1^ corresponds to the symmetric stretching vibration (*ν*_*1*_) of the phosphate ion PO_4_^3−^, the phosphate bending vibrations (*ν*_2_) 426 cm^−1^, 450 cm^−1^ and (*ν*_*4*_) appear at and 593 cm^−1^ respectively, the non-symmetric stretch (*ν*_*3*_) at 1035 and 1069 cm^−1^. There is a wide band at 1069–1073 cm^−1^, indicating type B carbonate substitution in the bone specimen (carbonate substituting for phosphate in the apatite lattice). The band at 1003 cm^−1^ corresponds to phenylalanine and HPO_4_^2−^ ion. The important Raman collagen bands are the amide III 1000–1260 cm^−1^ and amide I at 1656 cm^−1^ bands, which arise largely from the collagen, and the CH_2_ peak at approximately 1450 cm^−1^ which is present in both collagenous and non-collagenous organic molecules. Figure 3b, c shows pure basic components of implanted biomaterial. The 1-3-β-d-glucan bands are as follows: 1465, 1371 and 1048 cm^−1^ are attributed to the presence of polysaccharides. Band at 891 cm^−1^ refers to the anomeric structure about the glycosidic bond and in particular the configuration of the main β, α and polysaccharides. Band at 428 cm^−1^ indicates the presence in the sample β-1,3-glucan; absence of a band at 950 cm^−1^ which is assigned to α-1,3-glucan demonstrates the presence of only one form of glucan in the composite [38]. The glucan bands at 1107 and 1392 cm^−1^ can also be assigned to the *ν* sym (COC) glycosidic and δ (CH_2_) bands, respectively. The bands of CHAP granules are described the same as the phosphorous bands.

**Fig. 4:**
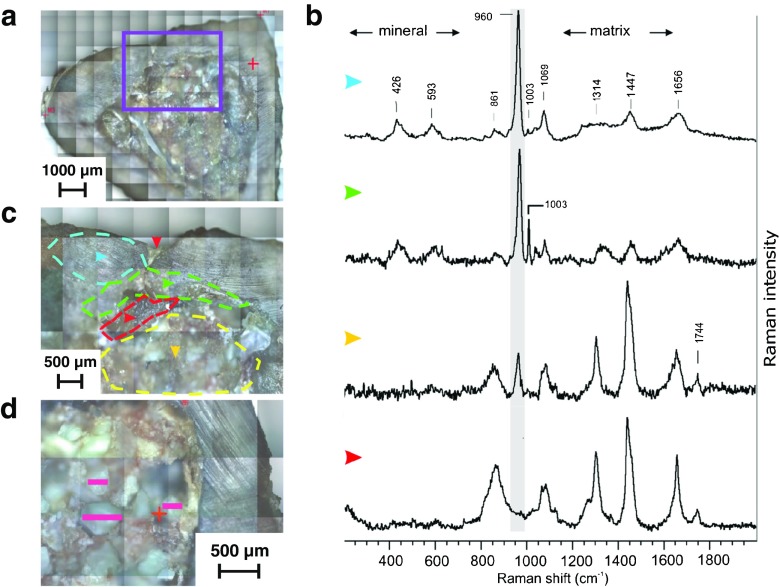
The composition of a bone section analysed by Raman spectroscopy. (**a**) Visible image of the 6-month sample; (**b**) the Raman spectra taken from points marked in the (**c**) image by *coloured arrowheads*: *blue arrowhead* compact bone, *green arrowhead* newly formed bone, *yellow arrowhead* implant remains, *red arrowhead* collagen and lipids; (**c**) magnified part of (**a**) sample with 4 marked areas discriminated on the basis of averaged Raman spectra; (**d**) the magnification of the opposed to drilling part of the sample. The *pink lines* corresponds to the CHAP granules size, the *short ones* are 250 μm and *long one* is 500 μm

The connection of optical microscope with Raman spectrometer enabled to distinguish several regions in bone samples with different spectra: compact bone, newly formed bone tissue, implant remains and region enriched with collagen and lipids (Fig. 4). In Fig. 4b, blue arrowhead indicates averaged Raman spectrum taken from the outer layer of a sample marked in Fig. 4c as blue area. This spectrum was assigned to a cortical bone as it exhibits the complementary bands as bone tissue in Fig. 3a. The spectrum of adjacent (green) area exhibits lower phosphorus bands and higher band at 1003 cm^−1^ and was attributed to newly formed bone tissue (Fig. 4b, c). The yellow area shows the implant remains with much lower phosphorus bands and high organic bands, which is especially characteristic for collagen and lipids. The comparison of the chemical changes in composite before (Fig. 3b, c) and after implantation is presented. It can be noted that the part between the newly formed bone and implant remains (which is marked with red arrow and red data set) do not have phosphorous bands, rather bands that are assigned for collagen and lipids at 1744 cm^−1^ (Fig. 4c). It was reported that the changes of lipid content in bone affect the metabolism of lipids, as the lipid content in articular cartilage increased with the age of the samples [39, 40]. Additional Raman images were presented on Fig. S1 the Electronic Supplementary Material (ESM) and exemplary differentiation between spectra within the selected area (red zone) were also presented on Fig. S2 in the ESM.

The optical microscope connected with Raman spectrometer enabled also to observe the bioresorption/dissolution of apatite. The composites were prepared using a mixture of particles with a size between 200 and 600 μm. As presented in Fig. 4d, the measured size of the largest remaining CHAP granules 6 months after composite implantation decreased to 500 μm. The smaller granules ca. 100 μm are visible and also smaller pieces, but due to the procedure of cutting, they may come from the large granules. Nevertheless, this result indicates degradation of granules localized inside the bone tissue.

**Fig. 5:**
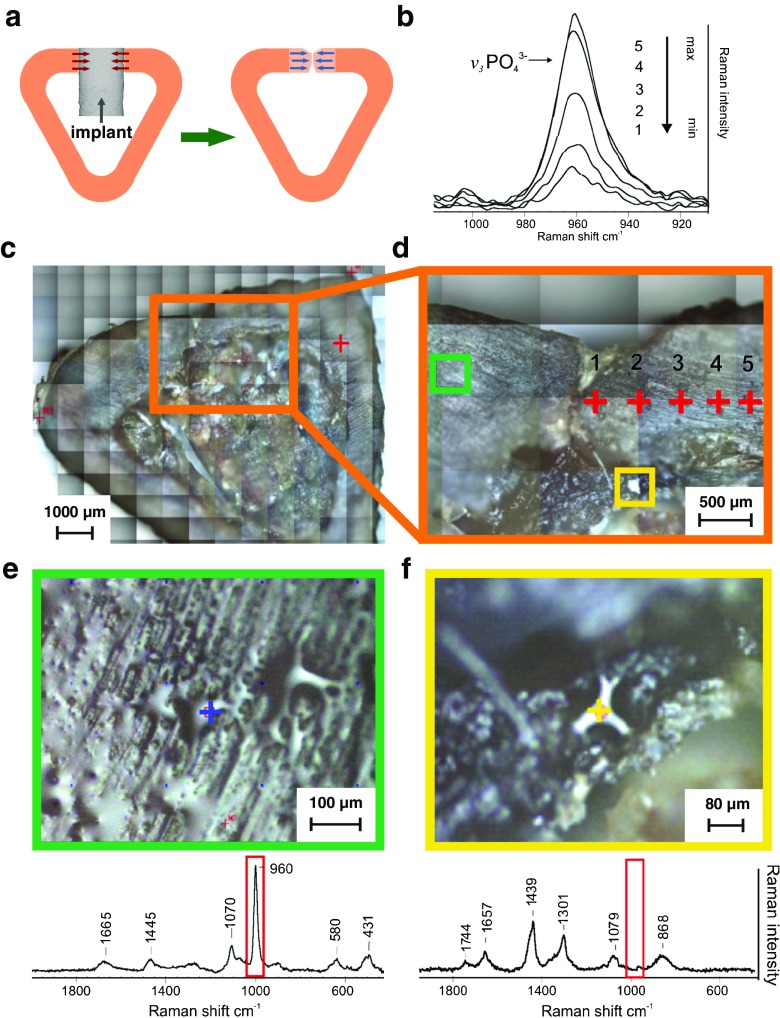
The defect area of rabbit tibia after implantation of CHAP/glucan composite at 6 months after surgery investigated by Raman spectroscope coupled with optical microscope. (**a**) Scheme of bone cross-section showing the direction of bone ingrowth into implant (*red arrows*) and the direction of mineralization process within newly formed bone (*blue arrows*); (**b**) the relative intensity of Raman spectra obtained from 5 different points; (**c**) visible image of bone cross-section with a narrow gap in cortical bone indicating the defect area; (**d**) magnified part of (**c**) sample including healed defect with marked 5 points; (**e**) the magnification of green square marked in the (**d**) image with corresponding Raman spectrum. (**f**) The magnification of the yellow square marked in the (**d**) image with corresponding Raman spectrum

Two important factors of bone quality are collagen crosslinking and the degree of mineralization [41]. The mineralization is a complex process involving bone cells (osteoblasts), growth factors (such as PDGF, TGF-β, IGF-I, IGF-II, FGF), endothelin (ET-1), bone morphogenetic proteins (BMPs) and others. As a result, crystals of calcium phosphate are produced and deposited within the bone’s fibrous matrix. Generally, this process implies two successive steps: a primary mineral deposition on the calcification front and subsequent slow process of secondary mineralization of basic structure units (BSUs) [42, 43]. The degree of secondary mineralization depends on the lifespan regulated by rate of turnover [44]. Bone tissue may exhibit a heterogeneous degree of mineralization in different areas; therefore, the properties of newly formed bone may differ in tissue maturity from older bone [35, 42]. Our study of the relative intensity of PO_4_^3−^ at 960 cm^−1^ in bone cross-sections revealed the differences in the phosphate content (Fig. 5b). Based on the obtained results, the scheme showing the direction of new bone formation and mineralization process was proposed (Fig. 5a). The spectra from marked points numbered 1–5 in the Fig. 5d revealed the highest phosphorus content in the point number 5 and the lowest in the point number 1. Other bands obtained from this point were illustrated in Fig. S3 in the ESM. The proportion between spectra of amides bands was the same as for phosphate bands. The distance between each point was 300 μm, and the total distance was about 1500 μm. The Raman intensity of phosphate bands confirms that this particular part of the sample is newly formed bone undergoing mineralization process. The difference of phosphorus content exhibit maturity of the bone tissue, the further away from the defect centre the higher mineralization/maturity of bone tissue. Therefore, the Raman spectroscopy may be a useful tool for estimating the degree of mineralization process and maturity of bone tissue.

**Fig. 6:**
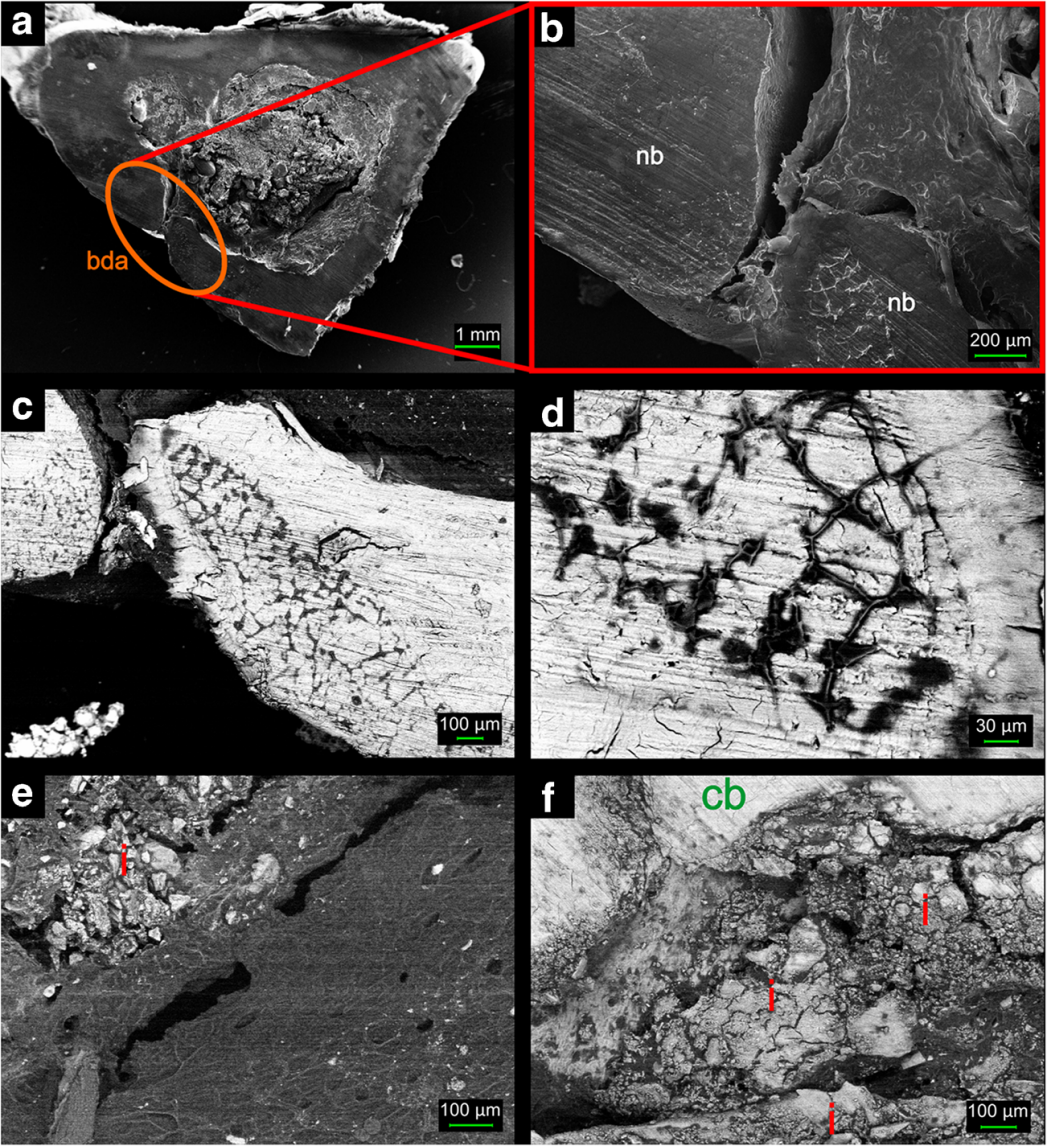
SEM images showing cross-section of non-decalcified bone 6 months after implantation. (**a**) General appearance with indicated 4 mm defect area—marked with *red circle*; (**b**–**d**) initial defect recovered with new bone tissue, visible ca. 100 μm cranny in the middle of defect and organic substance surrounded by mineralized bone; (**e**) implant debris in the *upper left corner* connected with new bone tissue; (**f**) compact bone tightly adhered to CHAP granules. Symbols: *bda* bone defect area, *nb* new bone tissue, *i* implant, *cb* compact bone

Figure 5e presented part of sample marked with the green square on Fig. 5d. This image is equivalent with the SEM images presented in Fig. 6c, d. The spectrum obtained from fibrous connector visible within presented area exhibit similarity to collagen type I (bands at 1665, 1445 and 1070 cm^−1^) together with phosphate bands at 960, 580 and 431 cm^−1^. It is the same as the representative spectrum of bone tissue (Fig. 3a) and indicate mineralization process of bone matrix. The yellow square marked in Fig. 5d and its magnification (Fig. 5f) was taken at the border between the bone tissue and remains of the implanted composite. The chemical characterization of visible fibrous connector indicates area enriched with collagen without phosphate bands. This exposes the mineralization process that has not started yet in this area and confirms that the CHAP/β-1,3-glucan composite is a suitable background for collagen ingrowth, as it significantly aids an increase in its maturity with the time of bone growth process.

Figure 6 shows the SEM images presenting elaborately the defect 6 months after composite implantation. In the triangle cross-section of the tibia (Fig. 6a), there is visible ca. 100 μm gap within compact bone that remained after regeneration of 4 mm drilled bone void. Figure 6b–d demonstrates that the new bone tissue in the defect area consists of organic matrix and mineralized bone, thus suggesting that the regeneration/mineralization process has not finished yet. Implant debris visible in the proximity of bone defect were connected with new bone tissue, as presented in Fig. 6e, f. Some CHAP granules were tightly entwined by bone matrix, and some were encapsulated by the mineralized bone; however, the most granules are situated in the bone implant border. The bone implant integration indicates good biocompatibility of the composite material.

**Fig. 7:**
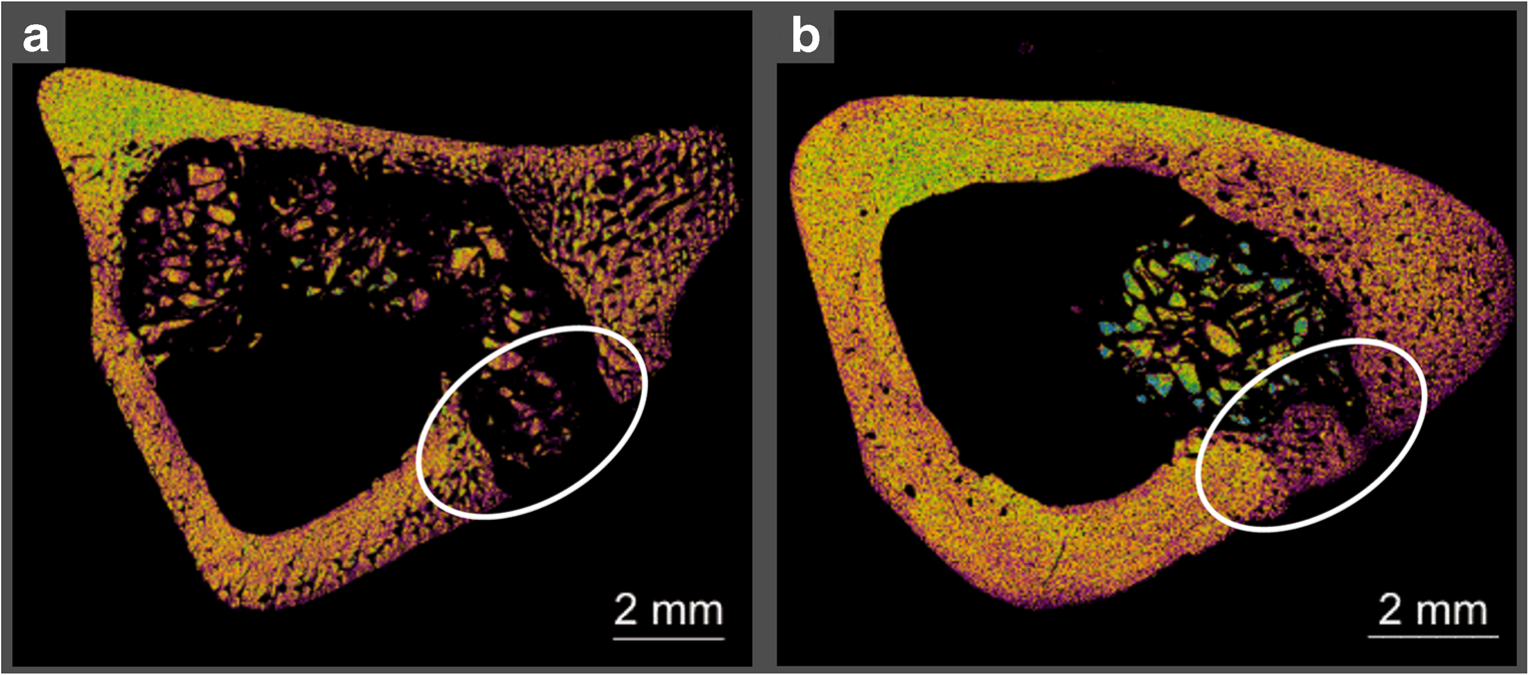
MicroCT images demonstrate integration of CHAP/glucan composite with the bone tissue after 1 month (**a**) and 3 months (**b**)

MicroCT images shown in Fig. 7 revealed that the new bone significantly recovered the defect area (in white circle) after 3 months. Implant debris were still visible around the bone defect site. Some composite particles were still embedded in compact bone; however, they appeared significantly smaller than others, indicating proceeding bioresorption (as presented also in Fig. 4d). The entire implants were highly radiopaque due to the dense structure of CHAP granules; thus, they were clearly visible in the area of implantation. Panel B exposed the penetration of newly formed osseous tissue into the 3-dimensional structure of the implant. Close contact between living bone and synthetic biomaterial and no signs of graft rejection were displayed. The results obtained by use of SEM (Fig. 6) and microCT (Fig. 7) are in accordance with the bone healing mechanism and compatible with the scheme presenting the direction of bone ingrowth/mineralization (Fig. 5a).

## Conclusions

In this study, we investigated in vivo the bioactivity of β-1,3-glucan/CHAP composite using Raman spectroscopy and different imaging techniques (SEM, microCT). The imaging tools such as optical microscope, SEM and high-resolution microCT showed relevant integration of ceramic-polymer composite material with bone tissue 6 months after implantation. Raman microspectroscopy has been employed to study chemical changes in the composite and material composition in bone. This analytical spectroscopic technique offers the possibility to obtain micro-level spatial resolution and permits the detection of local variations in composition. Based on this technique, bone regeneration in terms of (collagen rich) matrix formation and further mineralization was observed and described. Organic fibrils visible in the newly formed bone and at the bone implant border were assigned to collagen type I based on the spectrum of standard protein. It shows that the composite may serve as a biocompatible background for collagen ingrowth. The mineralization of newly formed bone reflected its maturation which progressed from the edge to the centre of defect area. Raman spectroscopy appeared to be very useful in estimating the degree of mineralization process and maturity of bone tissue. Implant debris were still visible after 6 months and remained inside the marrow cavity. This study leads to the conclusion that CHAP/glucan composite demonstrates bioactive and biocompatible properties for bone repair process.

The novel aspect of our approach is that we used complementary analytical and imaging techniques that provided a highly informative and reliable characterization of biological samples. We used Raman spectroscopy, microCT and SEM in the characterization of non-decalcified bone samples which requires minimal sample preparation process. This methodology illustrates the benefits of Raman spectroscopy in combination with microCT and high-resolution electron microscopy that allowed looking at the samples surface from a distance of different perspectives.

## Electronic supplementary material

ESM 1 (PDF 744 kb).

## Abbreviations

BGS: Bone grafts and substitutes
BMC: Bone mineral content
BMD: Bone mineral density
BMP: Bone morphogenetic protein
BSU: Basic structure unit
CHAP: Carbonate hydroxyapatite
ET: Endothelin
FGF: Fibroblast growth factor
IGF: Insulin-like growth factor
microCT: Microcomputed tomography
PDGF: Platelet-derived growth factor
SEM: Scanning electron microscope
TGF: Transforming growth factor
