# Supplementary material for: New approach in evaluation of ceramic-polymer composite bioactivity and biocompatibility
Source: Anal Bioanal Chem. 2017 Jul 26;409(24):5747–55. doi: 10.1007/s00216-017-0518-0 (PMC5583273; doi:10.1007/s00216-017-0518-0)
Supplement: Supplementary file 1 — (PDF 744 kb). [file 216_2017_518_MOESM1_ESM.pdf]

Analytical and Bioanalytical Chemistry

Electronic Supplementary Material

New approach in evaluation of ceramic-polymer composite bioactivity and biocompatibility

Leszek Borkowski, Anna Sroka-Bartnicka, Izabela Polkowska, Marta Pawlowska, Krzysztof Palka, Emil Zieba, Anna Slosarczyk, Krzysztof Jozwiak, Grazyna Ginalska

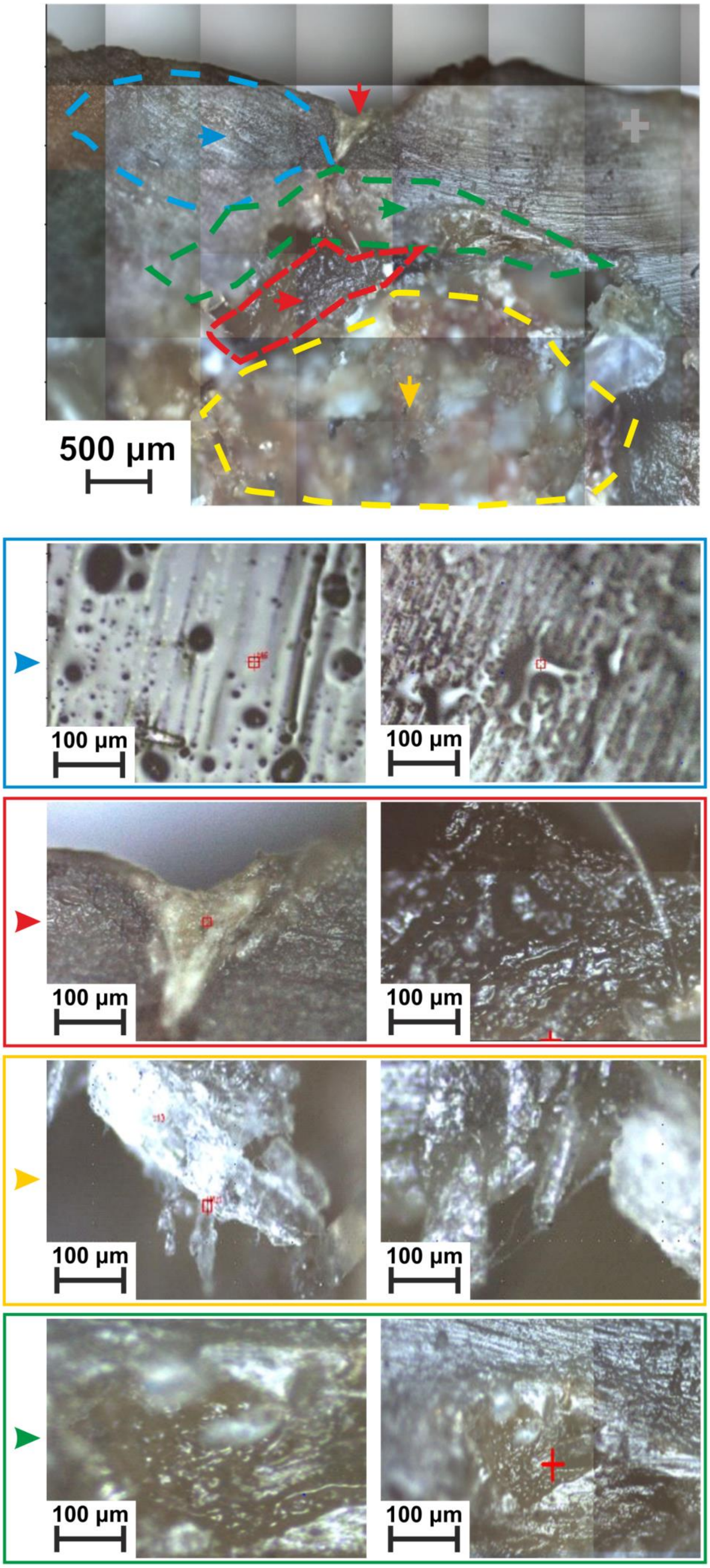

Fig. S1 Raman images showing heterogeneity of a sample in different zones

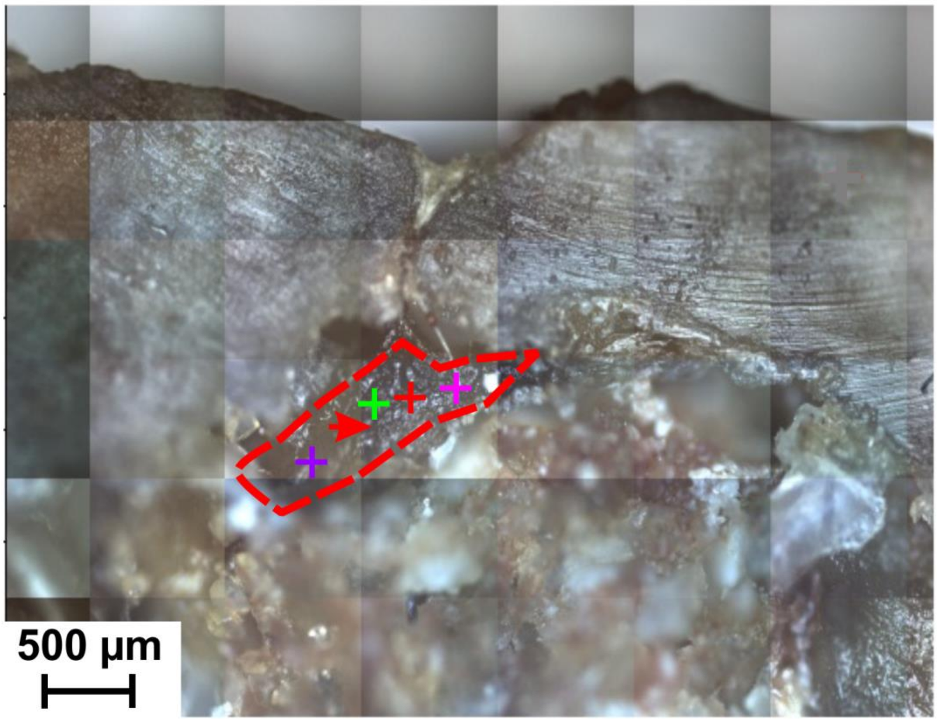

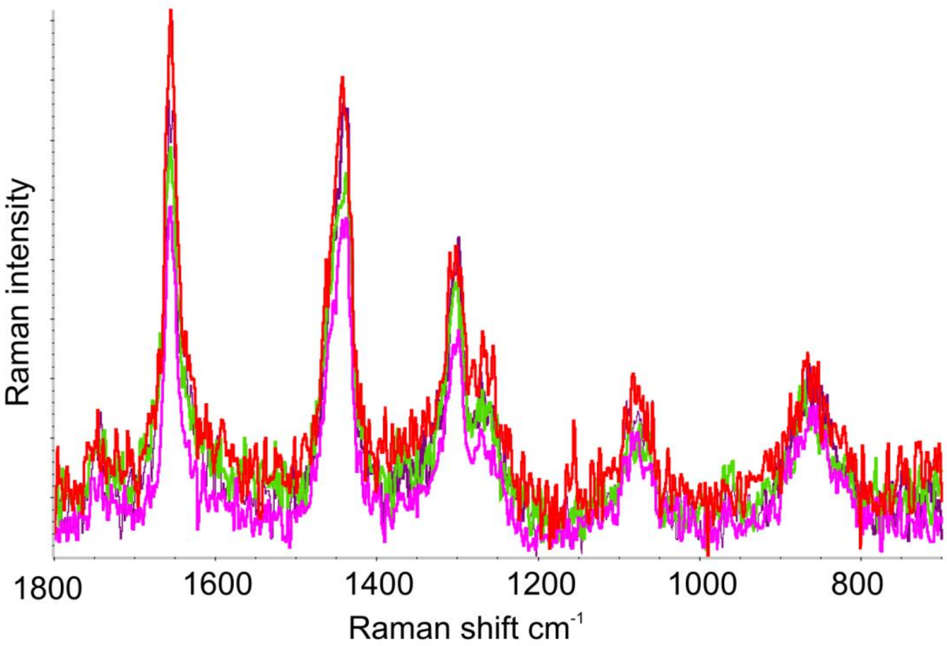

Fig. S2 The variations of the Raman spectra within the selected area (red zone in the image). The spectra were collected from marked points, were baseline corrected and show relative intensities of bands

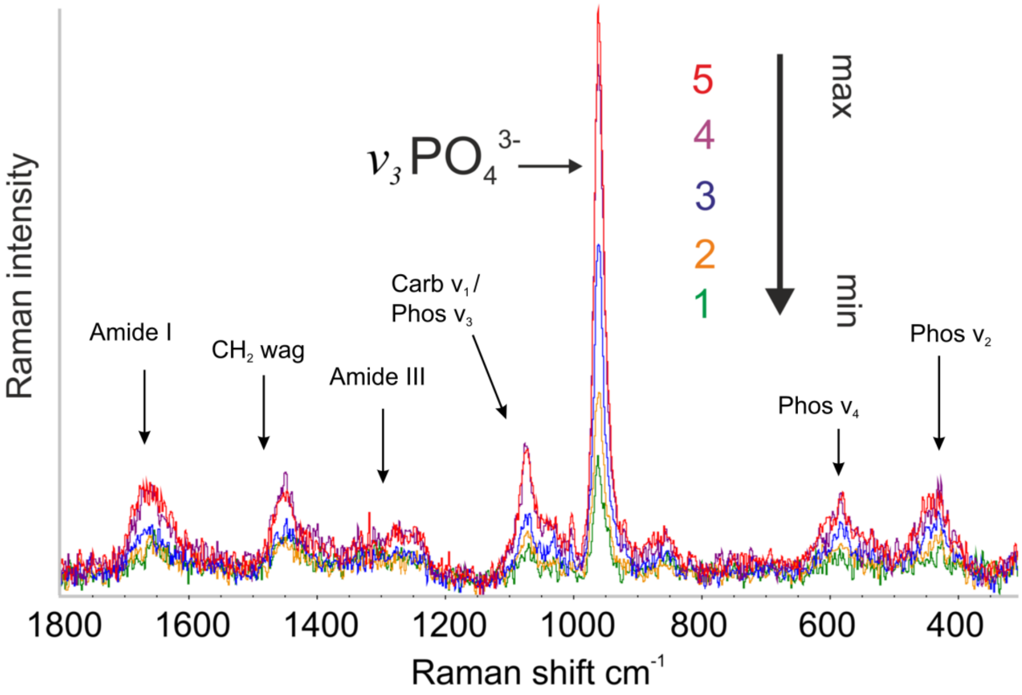

Fig. S3 The relative intesity of Raman spectra obtained from 5 difrent points in the measured range presented in Fig. 5b
